# Prenatal Maternal Stress Causes Preterm Birth and Affects Neonatal Adaptive Immunity in Mice

**DOI:** 10.3389/fimmu.2020.00254

**Published:** 2020-02-26

**Authors:** Valeria Garcia-Flores, Roberto Romero, Amy-Eunice Furcron, Dustyn Levenson, Jose Galaz, Chengrui Zou, Sonia S. Hassan, Chaur-Dong Hsu, David Olson, Gerlinde A. S. Metz, Nardhy Gomez-Lopez

**Affiliations:** ^1^Perinatology Research Branch, Division of Obstetrics and Maternal-Fetal Medicine, Division of Intramural Research, Eunice Kennedy Shriver National Institute of Child Health and Human Development, National Institutes of Health, U. S. Department of Health and Human Services, Detroit, MI, United States; ^2^Department of Obstetrics and Gynecology, Wayne State University School of Medicine, Detroit, MI, United States; ^3^Department of Obstetrics and Gynecology, University of Michigan, Ann Arbor, MI, United States; ^4^Department of Epidemiology and Biostatistics, Michigan State University, East Lansing, MI, United States; ^5^Center for Molecular Medicine and Genetics, Wayne State University, Detroit, MI, United States; ^6^Detroit Medical Center, Detroit, MI, United States; ^7^Department of Obstetrics and Gynecology, Florida International University, Miami, FL, United States; ^8^Office of Women's Health, Integrative Biosciences Center, Wayne State University, Detroit, MI, United States; ^9^Department of Physiology, Wayne State University School of Medicine, Detroit, MI, United States; ^10^Department of Obstetrics and Gynecology, Pediatrics, and Physiology, University of Alberta, Edmonton, AB, Canada; ^11^Department of Neuroscience, Canadian Centre for Behavioural Neuroscience, University of Lethbridge, Lethbridge, AB, Canada; ^12^Department of Immunology, Microbiology and Biochemistry, Wayne State University School of Medicine, Detroit, MI, United States

**Keywords:** preterm labor, neonates, offspring, birthweight, T cells

## Abstract

Maternal stress is a well-established risk factor for preterm birth and has been associated with adverse neonatal outcomes in the first and subsequent generations, including increased susceptibility to disease and lasting immunological changes. However, a causal link between prenatal maternal stress and preterm birth, as well as compromised neonatal immunity, has yet to be established. To fill this gap in knowledge, we used a murine model of prenatal maternal stress across three generations and high-dimensional flow cytometry to evaluate neonatal adaptive immunity. We report that recurrent prenatal maternal stress induced preterm birth in the first and second filial generations and negatively impacted early neonatal growth. Strikingly, prenatal maternal stress induced a systematic reduction in T cells and B cells, the former including regulatory CD4+ T cells as well as IL-4- and IL-17A-producing T cells, in the second generation. Yet, neonatal adaptive immunity gained resilience against prenatal maternal stress by the third generation. We also show that the rate of prenatal maternal stress-induced preterm birth can be reduced upon cessation of stress, though neonatal growth impairments persisted. These findings provide evidence that prenatal maternal stress causes preterm birth and affects neonatal immunity across generations, adverse effects that can be ameliorated upon cessation.

## Introduction

Stress can best be understood as the inability to adapt to environmental demands, namely acute and chronic stressors, and is known to cause adverse health outcomes (1). These demands range from traumas to daily nuisances, and the degree of experienced stress varies based on genetic, regulatory, and social factors (1). The underlying physiology of stress is well-understood by way of the hypothalamic-pituitary-adrenal (HPA) axis, ultimately leading to the secretion of glucocorticoids into the blood stream (2). Pregnant women are particularly vulnerable to stress given homeostatic adaptations during this period (3). Indeed, maternal stress is a well-established risk factor for preterm birth (4), the leading cause of perinatal morbidity and mortality worldwide (5, 6). Prenatal maternal stress has also been associated with physiological, neurological, and psychological consequences in the offspring (7–14).

The intra-uterine period is a window of vulnerability in the development of the fetal immune system (15). Hence, prenatal maternal stress is associated with increased susceptibility to disease and lasting immunological changes in the offspring (16, 17). Previous reports have shown that prenatal maternal stress contributes to an increased risk of immune-related disorders such as asthma (18) and allergies (19, 20) in children. Several potential and non-exclusive mechanisms whereby prenatal maternal stress induces adverse neonatal outcomes have been suggested, including epigenetic alterations (21) and dysregulation of the maternal/fetal HPA axis (22). Previous descriptive studies have also suggested that maternal stress impacts neonatal immunity (23). However, the mechanisms underlying the effects of prenatal maternal stress on neonatal adaptive immunity are poorly understood.

The deleterious consequences of prenatal maternal stress not only affect the first generation of newborns, but also may be transmitted across subsequent generations (24, 25). In fact, recent animal studies have shown that gestational stress across generations has downstream effects on the endocrine and metabolic pathways (26, 27). Importantly, intergenerational maternal stress gradually shortens the length of gestation (26) and affects physiological and molecular processes in both the mother and offspring (28).

In the current study, we used mice to evaluate the adverse effects of prenatal maternal stress on the timing of delivery across three generations. In addition, we performed deep immunophenotyping of the neonatal adaptive immune system to determine the lasting adverse effects of prenatal maternal stress across generations. Lastly, we evaluated whether the cessation of stress reverts the adverse pregnancy and neonatal outcomes induced by prenatal maternal stress.

## Materials and Methods

### Mice

C57BL/6 mice were purchased from The Jackson Laboratory (Bar Harbor, ME, USA), bred in the animal care facility at the C.S. Mott Center for Human Growth and Development, Wayne State University, Detroit, MI, and housed under a circadian cycle (light/dark = 12:12 h). Several generations were inbred in our animal facility prior to initiating the study. Dams between 8 and 12 weeks of age were mated with males of proven fertility, also 8 to 12 weeks of age; the dams were checked between 8:00 and 9:00 a.m. daily for the appearance of a vaginal plug, indicating 0.5 days post coitum (dpc), at which point female mice were removed from the mating cages and housed separately. Pregnancy was confirmed by a weight gain of ≥2 g at 12.5 dpc. All mouse experiments were approved by the Institutional Animal Care and Use Committee at Wayne State University (Protocol No. A-09-08-12 and A-07-03-15). The authors adhered to the National Institutes of Health Guide for the Care and Use of Laboratory Animals.

### Murine Model of Stress During Pregnancy Across Generations

**Figure 1:**
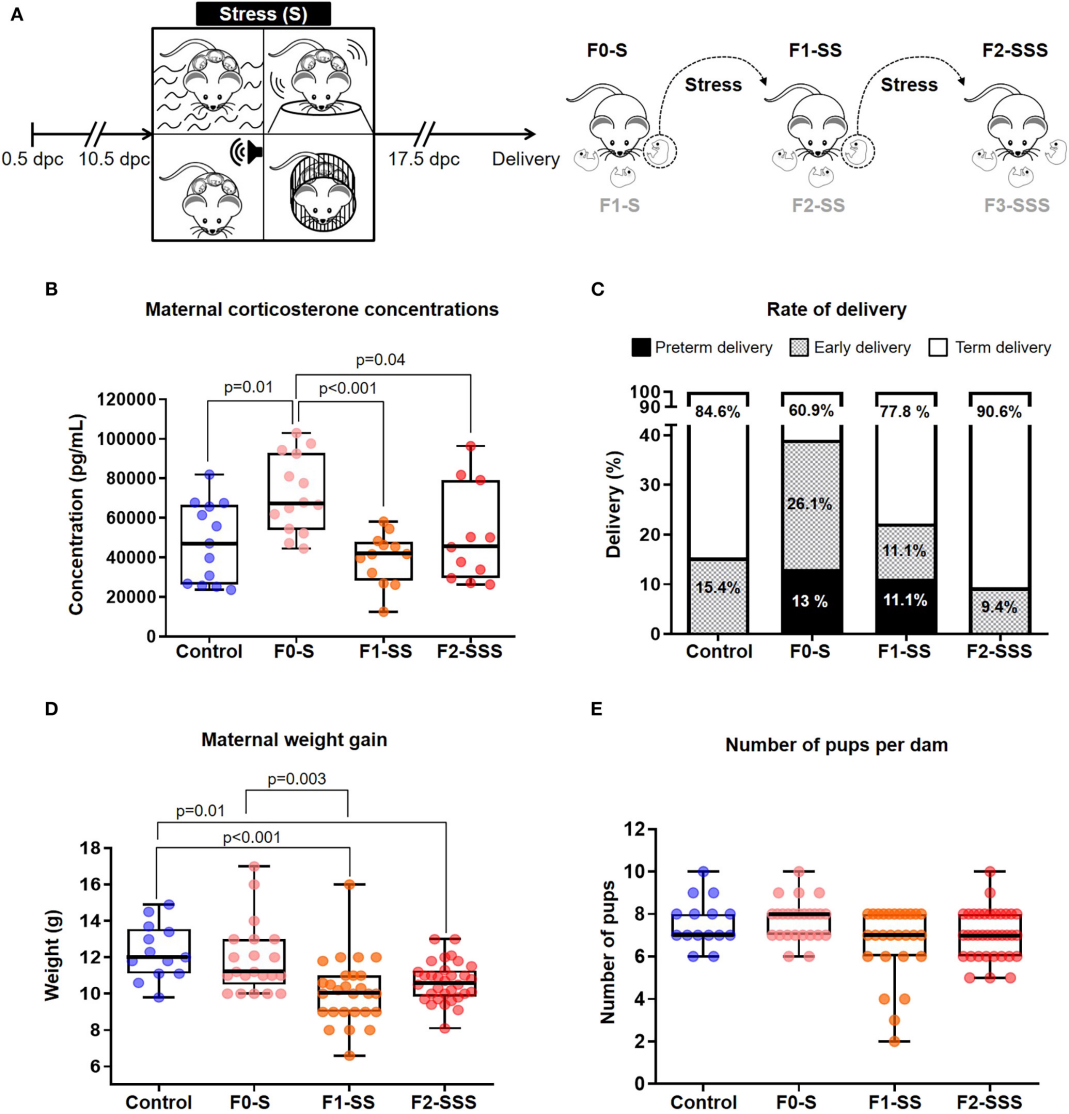
Maternal outcomes of prenatal maternal stress across generations. **(A)** Experimental design of alternating, unpredictable stress procedure. **(B)** Maternal corticosterone levels across generations 1 week after delivery (control *n* = 13; F0-S *n* = 14; F1-SS *n* = 12; F2-SSS *n* = 11). **(C)** Rates of delivery in each generation, specified as preterm, early, and term delivery (control *n* = 13; F0-S *n* = 23; F1-SS *n* = 27; F2-SSS *n* = 32). **(D)** Maternal weight gain across generations (control *n* = 13; F0-S *n* = 21; F1-SS *n* = 27; F2-SSS *n* = 32). **(E)** Number of pups per dam across generations (control *n* = 15; F0-S *n* = 25; F1-SS *n* = 27; F2-SSS *n* = 36). For box plots, mid-lines indicate medians, boxes indicate interquartile ranges, and whiskers indicate min-max range.

Three generations of mice were bred (Figure 1A) and divided into several groups: (1) Non-stressed controls; (2) Female mice stressed during gestation (F0-S); (3) Pregnant daughters stressed during gestation (F1-SS); and (4) pregnant granddaughters stressed during gestation (F2-SSS). Females from each litter were used to breed subsequent generations, and each generation was composed of subjects from several different mothers and fathers. Three different cohorts of mice were utilized in this study; the first two to obtain observational data in two different years (2013 and 2014) and the third to determine maternal corticosterone levels and perform immunophenotyping of the neonates (2014–2015). Each group, including the control group, refers to an amalgamation of these cohorts. It is worth mentioning that we did not include one control group for every generation because we used in-bred mice under consistent conditions and followed the laws that mandate replacement alternatives, reduction alternatives, and refinement alternatives (The Three R's) in scientific research (29).

### Schedule of Prenatal Stressors

Prenatal stress was applied daily from 10.5 to 17.5 dpc without interruption. The four stress procedures were adapted from well-established rodent stress models and included swimming (30, 31), restraint (30, 31), shaking (32), and white noise (33). Two of the four procedures were applied daily in an alternating, unpredictable sequence from 8:00 a.m. to 4:00 p.m. Stress treatments were performed in a designated room other than the housing facility. For the swimming procedure, a small tub was filled with room temperature water (~22°C). Mice were individually placed in the tub and made to swim for 5 min (with a rest of 10 s). The water was deep enough to prevent the feet or tail from contacting the bottom of the tub (*Link to* Supplementary Video 1). For the restraint procedure, mice were placed inside of a Plexiglas restrainer (2.5 cm inner diameter) in a standing position without compression of the body for a period of 20 min. The container had perforated ends to allow ventilation (*Link to* Supplementary Video 2). For the shaking procedure, mice were placed inside of a transparent, plastic 1-liter beaker and held on a low-speed vortex (speed 9) for a period of 5 min (*Link to* Supplementary Video 3). For the white noise procedure, mice were placed in a clean cage free of bedding. The cage was placed in a fume hood with a high-speed fan faced away from the cage generating white noise for 20 min (*Link to* Supplementary Video 4). Following the stress procedure on 16.5 dpc, dams were placed under recording *via* video camera (Sony Corporation, Tokyo, Japan) until delivery to evaluate maternal and neonatal outcomes. The number of feces produced during each procedure was recorded as a readout of stress response (Supplementary Figure 1).

### Maternal and Neonatal Outcome Variables

Gestational length was utilized as a pregnancy parameter, defined as the day mice were plugged (0.5 dpc) until the time the first pup was delivered. Based on the gestational length, the rate of delivery was categorized as (1) preterm delivery occurring 17–18.5 dpc; (2) early delivery occurring 18.5–19 dpc; and (3) term delivery occurring after 19 dpc. The rate of each delivery group was represented by the number of females delivering within that timeframe among the total number of mice within that generation. Maternal weight gain was also measured, defined as the total weight gained from the day mice were plugged until 16.5 dpc. The number of pups per dam was also recorded. Additionally, the occurrence of neonatal mortality at birth was recorded, defined as the number of pups born dead among the total litter size. After delivery, the mother and her pups were kept under observation and offspring weights were recorded 1, 2, and 3 weeks after birth.

### Maternal Corticosterone Determination

One week after delivery, dams (stressed or non-stressed controls) were euthanized to collect peripheral blood by intra-cardiac puncture between 9:00 and 10:00 a.m. The peripheral blood was centrifuged at 1,300 × g for 10 min at 4°C after collection, and serum was separated to be stored at −20°C until analysis. Total corticosterone concentrations were measured using a multiple species Cortisol Immunoassay Kit (Cat #IRAAKT2546, Innovative Research, Inc., Novi, MI, USA), according to the manufacturer's instructions. The colorimetric reaction was read using a programmable spectrophotometer (SpectraMax M5 Multi-Mode Microplate Reader, Molecular Devices, Sunnyvale, CA, USA). The assay sensitivity was 17.3 pg/mL, according to the manufacturer.

### Immunophenotyping of Neonates

Neonates born to stressed or non-stressed control dams were euthanized 1 week after birth and spleens were collected. The spleens were dissociated using glass slides and 1X phosphate-buffered saline (PBS), filtered using FACS buffer (BSA 0.1%, Sodium Azide 0.05%, and 1X PBS), and centrifuged at 1,300 × g for 10 min at 4°C to obtain a cell suspension and to perform immunophenotyping. The splenic cell suspensions were incubated with CD16/CD32 (FcγIII/II Receptor; BD Biosciences, San Jose, CA, USA) for 10 min, followed by extracellular and/or intracellular staining for immunophenotyping (Supplementary Table 1). For the staining of T cell and B cell populations, either the FoxP3 Staining Buffer Kit (Cat # 00-5523-00, eBiosciences, San Diego, CA, USA) or the Cytofix/Cytoperm Fixation/Permeabilization Solution (Cat# 554714, BD Biosciences) was used prior to intra-nuclear or intra-cellular staining, respectively. For the staining of CD71+ erythroid cells, the 1X FACS Lysing Solution (BD Biosciences) was used. Upon completion of the staining procedures, cell pellets were washed with 1X PBS and re-suspended in 0.5 mL FACS buffer. Samples were acquired using the BD LSRFortessa® Flow Cytometer (BD Biosciences) and analyzed with BD FACSDiva® Software Version 7.0 (BD Biosciences). The analysis and figures were performed using FlowJo software version 10 (FlowJo, LLC, Ashland, OR, USA). The absolute number of cells was determined using CountBright absolute counting beads (Molecular Probes, Eugene, OR, USA).

Immunophenotyping of T cell populations included the identification of naïve (CD3+CD44–CD62L+), memory (CD3+CD44+CD62L+), and effector (CD3+CD44+CD62L−) T cells [CD4+ and CD8+ naïve T cells (CD4+CD44−CD62L+; CD8+CD44−CD62L+, respectively), CD4+ and CD8+ memory T cells (CD4+CD44+CD62L+; CD8+CD44+CD62L+), and CD4+ and CD8+ effector T cells (CD4+CD44+CD62L−; CD8+CD44+CD62L−)]. We also identified the following T cell subsets: conventional T cells (CD3+), CD4+ T cells (CD3+CD4+), CD8+ T cells (CD3+CD8+), regulatory CD4+ T cells (CD3+CD4+CD25+FoxP3+), CD8+FoxP3+ cells (CD3+CD8+CD25+FoxP3+), Th1 cells (CD3+CD4+IFNγ+), Th2 cells (CD3+CD4+IL-4+), Th17 cells (CD3+CD4+IL-17A+), CD8+IL-17A+ T cells (CD3+CD8+IL-17A+), CD8+IFNγ+ cells (CD3+CD8+IFNγ+), and CD8+IL-4+ cells (CD3+CD8+IL-4+). B cell subsets were identified as: total B cells (B220+), B1-like cells (B220+CD5+), and B2-like cells (B220+CD23+). Lastly, CD71+ erythroid cells (CD3-CD71+TER119+) were also identified.

### Intergenerational Stress Cessation

In a third cohort, first-generation daughters born to prenatally stressed mothers were not subjected to the prenatal stress model during their pregnancies (F1-SNS). This cessation of intergenerational stress was done in comparison to mice from the same litter that continued to undergo the prenatal stress model. Maternal and neonatal outcomes were recorded *via* video camera and the weights of the offspring were recorded at weeks 1, 2, and 3.

### Statistical Analysis

**Figure 2:**
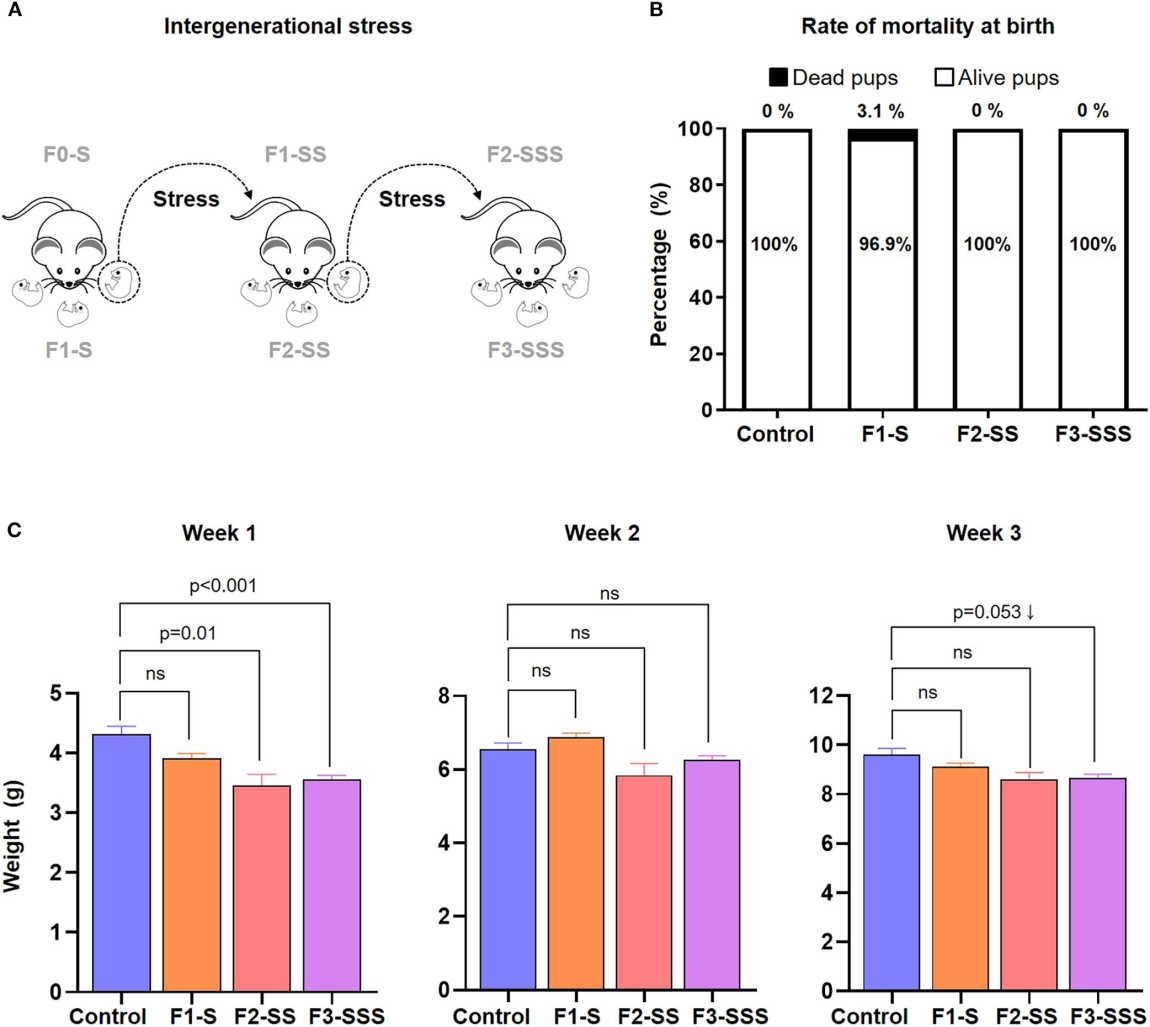
Neonatal outcomes of prenatal maternal stress across generations. **(A)** Experimental design of neonates born to stressed dams across generations. **(B)** Rate of neonatal mortality at birth in each generation (control *n* = 10; F1-S *n* = 19; F2-SS *n* = 19; F3-SSS *n* = 30). **(C)** Growth trajectory of neonates in the first 3 weeks after birth (control *n* = 5 litters; F1-S *n* = 8 litters; F2-SS *n* = 2 litters; F3-SSS *n* = 21 litters). Data are presented as mean ± standard error of the mean.

**Figure 3:**
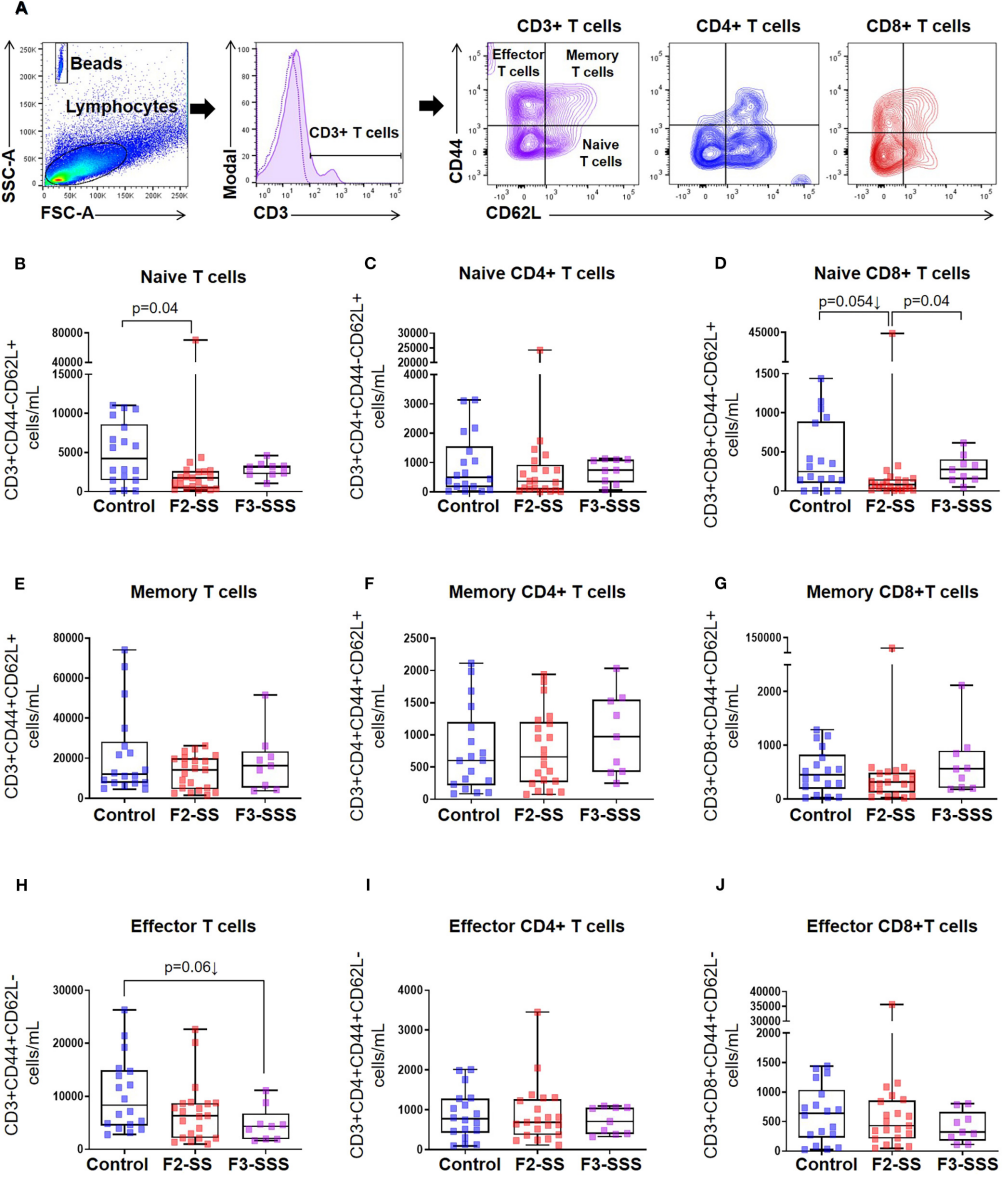
Immunophenotyping of naïve, memory, and effector T cells in prenatally stressed neonates. **(A)** Gating strategy used to identify T cell subsets. **(B–D)** Number of naïve T cells, **(E–G)** memory T cells, and **(H–J)** effector T cells (control *n* = 19; F2-SS *n* = 21; F3-SSS *n* = 9). Mid-lines indicate medians, boxes indicate interquartile ranges, and whiskers indicate min–max range.

**Figure 4:**
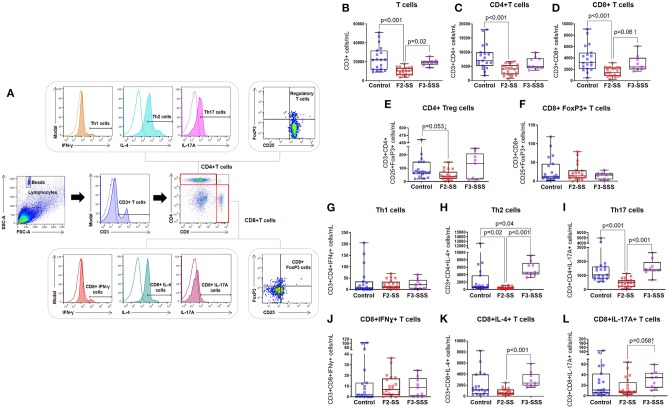
Immunophenotyping of T cell subsets in prenatally stressed neonates. **(A)** Gating strategy used to identify T cell subsets. **(B)** Number of conventional T cells, **(C)** CD4+ T cells, **(D)** CD8+ T cells, **(E)** regulatory CD4+ T cells or CD4+ Tregs, **(F)** CD8+FoxP3+ T cells, **(G)** Th1 cells, **(H)** Th2 cells, **(I)** Th17 cells, **(J)** CD8+IFNγ+ T cells, **(K)** CD8+IL-4+ T cells, **(L)** CD8+IL-17A+ T cells (control *n* = 19; F2-SS *n* = 21; F3-SSS *n* = 9). Mid-lines indicate medians, boxes indicate interquartile ranges, and whiskers indicate min–max range.

**Figure 5:**
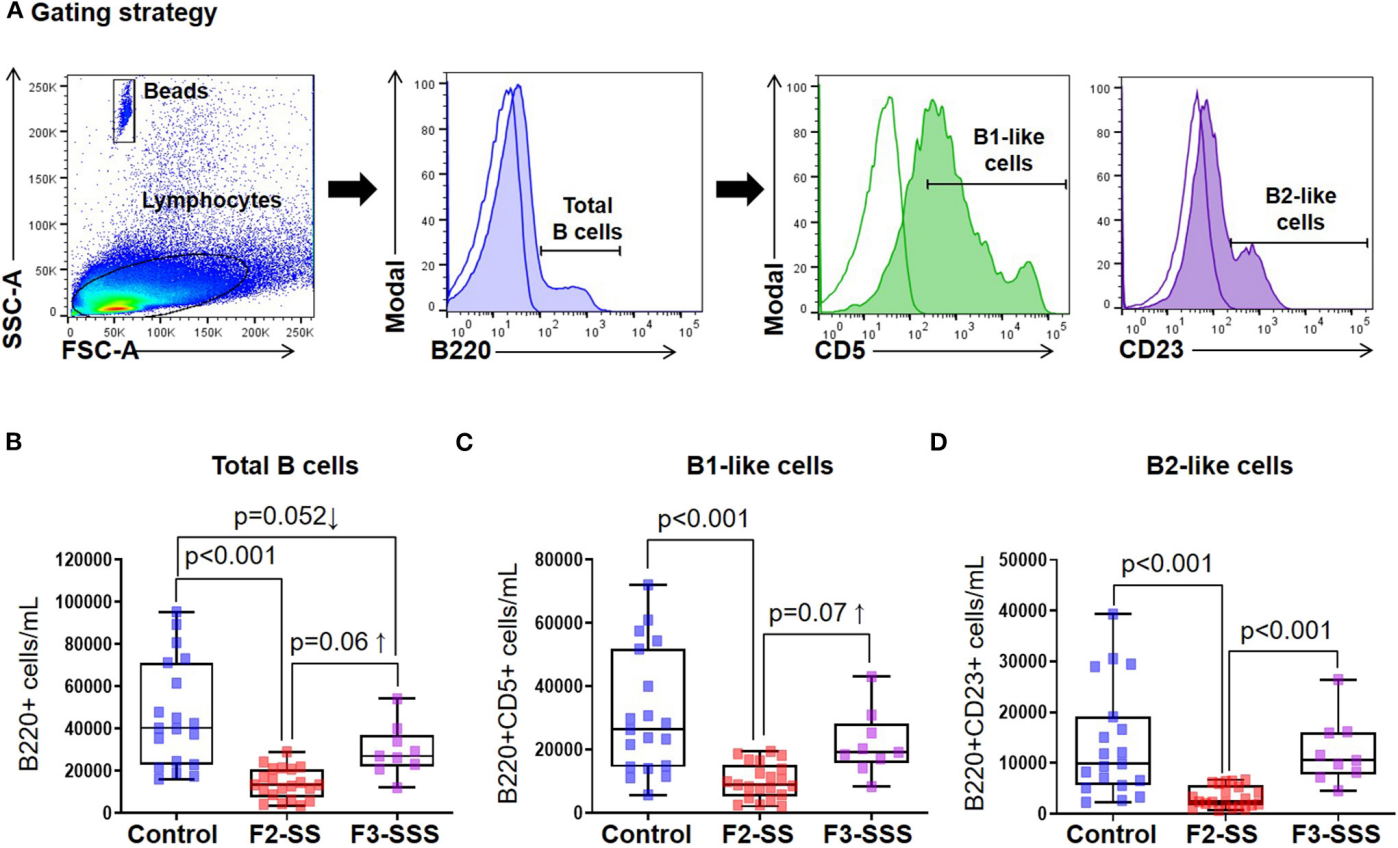
Immunophenotyping of B cells in prenatally stressed neonates. **(A)** Gating strategy used to identify B cells. **(B)** Total number of B cells, **(C)** B1-like cells, and **(D)** B2-like cells (control *n* = 19; F2-SS *n* = 21; F3-SSS *n* = 9). Mid-lines indicate medians, boxes indicate interquartile ranges, and whiskers indicate min–max range.

**Figure 6:**
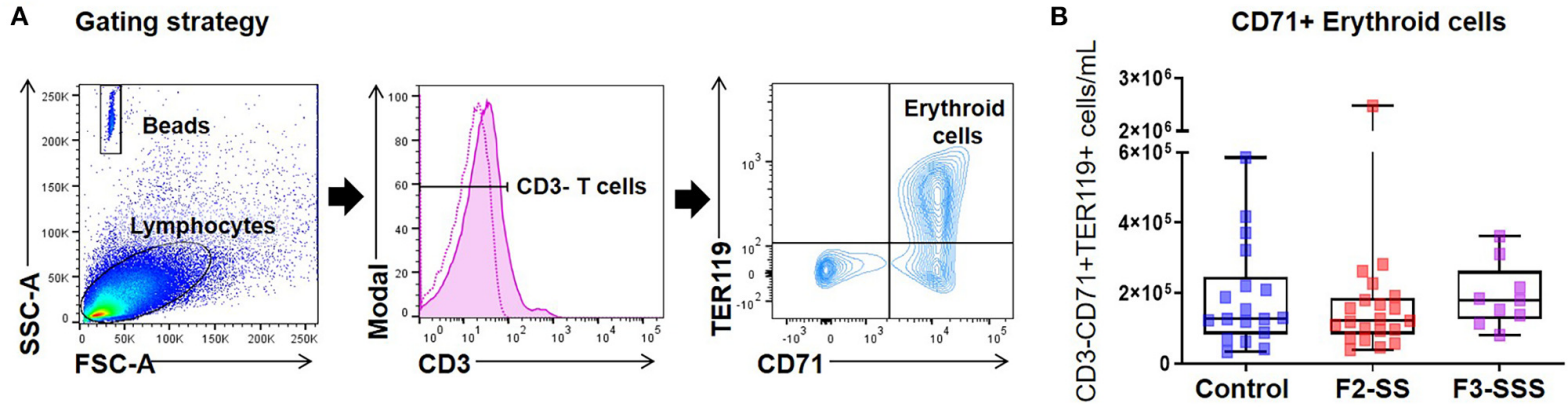
Immunophenotyping of CD71+ erythroid cells in prenatally stressed neonates. **(A)** Gating strategy used to identify CD71+ erythroid cells. **(B)** Number of CD71+ erythroid cells (control *n* = 18; F2-SS *n* = 21; F3-SSS *n* = 9). Mid-lines indicate medians, boxes indicate interquartile ranges, and whiskers indicate min–max range.

**Figure 7:**
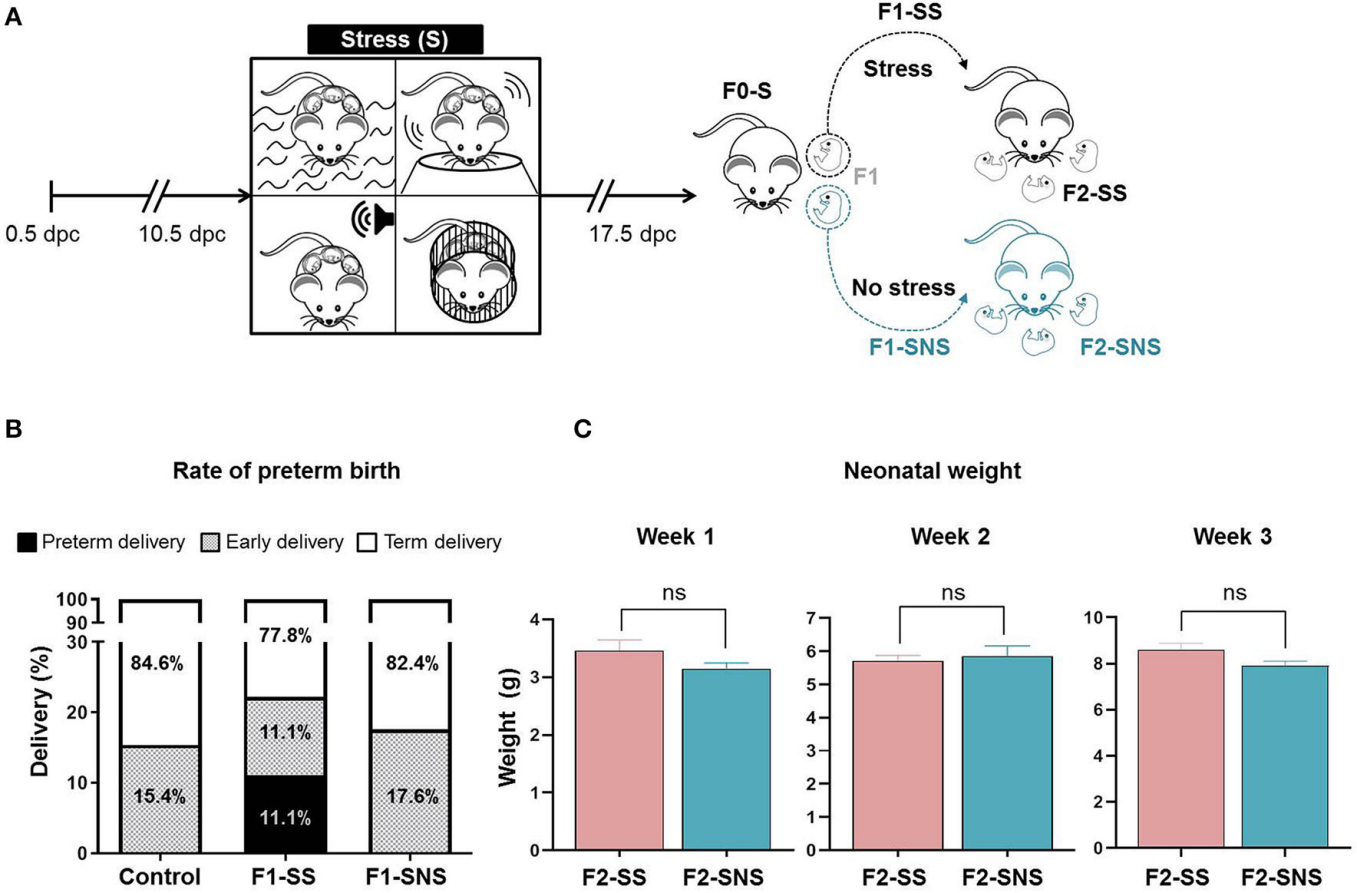
Experimental outcome of stress cessation cohort. **(A)** Experimental design of stress procedure. Animals of the parental generation (F0-S) were stressed and their pregnant daughters were either stressed (F1-SS) or not stressed (F1-SNS). **(B)** Rates of delivery in the control, F1-SS, and F1-SNS groups (control *n* = 13; F1-SS *n* = 27; F1-SNS *n* = 17). **(C)** Growth trajectory of F2-SS and F2-SNS neonates in the first 3 weeks after birth (F2-SS *n* = 2 litters; F2-SNS *n* = 10 litters). Data are presented as mean ± standard error of the mean.

Data were analyzed using SPSS Statistics Software Version 19.0, 2010 (IBM, Armonk, NY, USA). A Shapiro–Wilk test was performed to determine whether the data was normally distributed. For Figures 1–**6**, multiple comparisons were performed using ANOVA or Kruskal-Wallis tests with corresponding *post-hoc* tests. For **Figure 7**, the two groups were compared using a Student's *t-*test. An adjusted *p* ≤ 0.05 was considered statistically significant.

## Results

### Prenatal Maternal Stress Induces Preterm Birth in a Subset of Dams

First, we created a model of multiple, unpredictable stressors that included swimming, shaking, white noise, and restraint procedures (Figure 1A). We validated the efficacy and lack of desensitization to our alternating model by fecal pellet quantification (Supplementary Figure 1), a well-established indicator of murine stress (34–36). Continuously marked fecal production was observed throughout the procedure between generations, suggesting an incessant stress response. To further corroborate our stress model, we quantified maternal corticosterone concentrations in serum, a known hormonal biomarker of stress (37). It was found that the first generation of dams (F0-S) had the highest corticosterone concentration compared to the second (F1-SS) and third (F2-SSS) generations (Figure 1B), suggesting that the first generation suffers from acute stress while the subsequent generations suffer from chronic stress.

Next, we investigated whether prenatal stress could induce preterm birth or early delivery. This research question was based on strong associations between stress and preterm delivery (38–44). We found that stress prompted preterm delivery and early delivery in the first and second generations (Figure 1C). Specifically, the rate of preterm birth in the first generation (F0-S) and second generation (F1-SS) was 13% (3/23) and 11.1% (3/27), respectively. Although some of the controls experienced early delivery, this rate was elevated in the first stressed generation (F0-S 26.1% 6/23 vs. controls 15.4% 2/13). The second (F1-SS) and third (F2-SSS) generations also displayed early delivery (F1-SS 11.1% 3/27 & F2-SSS 9.4% 3/32), but these rates were similar to controls (15.4% 2/13). These data show that prenatal maternal stress can induce preterm birth in the first and second generations; however, such an effect was not observed in the third generation.

Given that stress can cause a reduction in litter size and resorption of implanted embryos (33), we investigated whether prenatal stress impacts maternal weight gain across generations. Consistently, it was found that total weight gain successively decreased throughout the generations (Figure 1D), a likely consequence of the decreased numbers of pups per dam (Figure 1E, non-significant). The data provide supporting evidence showing that prenatal maternal stress affects overall reproductive health.

### Prenatal Maternal Stress Impairs Neonatal Growth

Next, we investigated the impact of prenatal maternal stress on the immediate and long-term health of the offspring (Figure 2A). We first measured the rate of mortality at birth and found no significant differences amongst the stressed groups compared to controls (Figure 2B). However, we continued to monitor the growth of the offspring and found that neonatal weight was significantly decreased in each generation within the first week of life (Figure 2C). A similar non-significant trend was consistently observed at weeks 2 and 3 (Figure 2C). Indeed, pups born to stressed dams seemed smaller compared to controls (data not shown). This data shows that while prenatal maternal stress does not induce neonatal mortality, it does impact neonatal growth in early life.

### Prenatal Maternal Stress Alters the Neonatal T Cell Repertoire

Thus far, our findings show that stress compromises early offspring growth development in the second (F1-SS) and third (F2-SSS) generations of stressed dams. Therefore, we further explored any potential immunological detriments in F2-SS and F3-SSS neonates, born, respectively, to F1-SS and F2-SSS stressed dams. To measure the effect of cumulative prenatal maternal stress on immunocompetence, we first quantified neonatal T cell populations using flow cytometry (Figure 3A). In general, T cells can be divided into naïve, effector, and memory populations; these states are acquired through early recognition of self- or non-self-antigens (e.g., early-life microbiota) (45–48). We found that naïve T cells and naïve CD8+ T cells were significantly reduced in the F2-SS generation, while a downward trend was seen in naïve CD4+ T cells (Figures 3B–D). This observed reduction was overcome in the F3-SSS generation (Figures 3B,D). The memory T cell population was not changed in either the F2-SS or F3-SSS generation compared to controls (Figures 3E–G). There were non-significant alterations in the total effector T cells (Figures 3H–J). These results show that prenatal maternal stress reduces the pool of neonatal naïve T cells in the second generation, but not in the third generation.

After observing an alteration in the naive T cells of neonates born to stressed dams, we further investigated the impact of prenatal maternal stress on T cell subsets in F2-SS and F3-SSS neonates, born, respectively, to F1-SS and F2-SSS stressed dams (Figure 4A). We first broadly looked at conventional T cells and found a reduction in T cells (Figure 4B), CD4+ helper T cells (Figure 4C), and CD8+ cytotoxic T cells (Figure 4D) in the F2-SS neonates compared to controls. These reductions were all overcome by the F3-SSS generation. Since regulatory T cells play a critical role in neonatal development (49–51), we investigated whether prenatal maternal stress alters such a T cell subset. Interestingly, we found that regulatory CD4+ T cells tended to decrease in F2-SS neonates compared to controls, but this effect was restored in the F3-SSS generation (Figure 4E). Given that CD8+ T cells also express the transcriptional factor FoxP3 and seem to have regulatory properties (52–54), we also investigated whether such cells were altered in neonates born to stressed dams. No significant differences were seen in CD8+ cells expressing FoxP3 (Figure 4F). Furthermore, we quantified the Th1, Th2, and Th17 cell subsets of these neonates. In the Th1 cell subset, no differences were observed between the F2-SS and F3-SSS groups compared to controls (Figure 4G). However, the Th2 and Th17 cell types were significantly reduced in the F2-SS generation, but these reductions were phenotypically overcome in the F3-SSS generation (Figures 4H,I). The Th2 and Th17 findings mirrored our data in CD8+ T cells expressing IL-4 or IL-17A (Figures 4K,L). However, no differences were seen in the cytotoxic T cells expressing IFNγ (Figure 4J). In summary, prenatal maternal stress causes a systematic reduction of CD4+ T cells and, to a lesser extent, CD8+ T cells, expressing the cytokines IL-4 and IL-17A in the second generation, but such effects are restored in the third generation.

### Prenatal Maternal Stress Reduces B Cell Subsets

In addition to T cells, neonatal immunocompetence requires the function of B cells, and given their immaturity, B cells are quite distinct from those present in adults (55). B cells are generally divided into two predominant subsets: B2 cells that circulate through the blood and secondary lymphoid tissues and respond to antigens (56), and B1 cells that produce IgM and IgA for protection against pathogens (57–59). Using flow cytometry, we investigated the total number of B cells as well as B1-like and B2-like cell subsets in neonates born to prenatally stressed dams or controls (Figure 5A). Total B cells and the evaluated B cell subsets were decreased in F2-SS neonates compared to controls (Figures 5B–D). Yet, there was a re-establishment of total and B cell subsets in the F3-SSS generation to values comparable to controls (Figures 5B–D). These results indicate that prenatal maternal stress causes a systematic reduction in the B cell repertoire, which is restored in the third generation.

### Prenatal Maternal Stress Does Not Alter Neonatal CD71+ Erythroid Cells

Growing evidence shows that neonatal immunity depends on the critical immunosuppressive function of CD71+ nucleated erythroid cells (60–65). Indeed, we have shown that cord blood CD71+ erythroid cells play a central role in the modulation of inflammatory responses of neonates, which may be defective in those delivered prematurely (66, 67). Therefore, we investigated whether prenatal maternal stress alters the number of CD71+ erythroid cells in neonates (Figure 6A). To our surprise, neonatal CD71+ erythroid cells were unchanged in the F2-SS and F3-SSS generations (Figure 6B). The data show that prenatal maternal stress does not affect the neonatal CD71+ erythroid cells in number, yet further studies are required to investigate whether maternal stress alters the functionality of such cells.

### Cessation of Prenatal Maternal Stress Can Reduce Preterm Birth Rate, but Does Not Restore Neonatal Growth Impairment

Lastly, we investigated the inheritability of stress-induced alterations and evaluated whether these consequences could be overcome by the cessation of stress. We separated the litter delivered by an F0-S mother into two groups: one group that continued with intergenerational stressors (F1-SS) and another group in which stress was ceased (F1-SNS) (Figure 7A). We then compared the rate of preterm birth between the stressed F1-SS group and cessation F1-SNS group. Importantly, the cessation of stress resulted in all dams delivering at term or having an early delivery, which is comparable to controls. Specifically, the rate of preterm birth in F1-SS dams was 11.1% (3/27) whereas no animals delivered preterm in the control and cessation groups (Figure 7B). The rate of early delivery in the cessation group, 17.6% (3/17), was similar to that of controls, 15.4% (2/13) (Figure 7B). We further examined the impact of the cessation of stress on neonatal growth and did not find any significant differences between the F2-SS and F2-SNS neonates, indicating that the weight reduction observed in neonates born to stressed dams (Figure 2C) was not recovered with cessation of stress (Figure 7C). The data show that prenatal maternal stress-induced preterm birth can be fully attenuated by the interruption of stressful stimuli in the second generation, yet neonatal growth may still be impacted.

## Discussion

The current study provides evidence that prenatal maternal stress: (1) induces preterm birth across the directly exposed first and second generations in mice, (2) impairs neonatal growth at weeks one, (3) reduces the number of neonatal conventional CD4+ and CD8+ T cells in the second and third generations, (4) alters the neonatal T-cell subsets (Th2 and Th17) in the second generation, but not in the third generation, (5) causes a systematic reduction in the number of neonatal B cells in the second generation, but not in the third generation, and (6) does not alter the number of neonatal CD71+ erythroid cells across generations. Importantly, we report that the cessation of prenatal maternal stress in the second generation can attenuate preterm birth; yet, this intervention does not restore neonatal growth impairment. These findings provide insights into the causal relationship between prenatal maternal stress and neonatal immunity, which is further discussed below.

Pregnancy itself can be classified as a stressor given that endocrine and immune adaptations, largely mediated by the placenta, must occur in order for the mother to support the developing fetus (15, 68). Under normal circumstances, the systemic levels of adrenocorticotropic hormone (ACTH) and corticotropin-releasing hormone (CRH) progressively rise throughout early gestation, followed by an exponential increase prior to parturition (69–71). External stressors can further activate the placental maternal pituitary-adrenal axis in a manner consistent with the classic endocrine response, leading to the production of glucocorticoids (72, 73). Therefore, external stressors are perceived to prematurely activate such a pathway, leading to preterm labor and birth (70–72, 74). Consistent with this hypothesis, in the current study, we report that systemic corticosterone levels were increased in dams stressed during their first pregnancy. However, corticosterone levels remained at their basal state during the second and third generations. This finding is consistent with the attenuation of cortisol increase that has been observed in both repeat exposures to stress, as well as in the offspring of stressed parents (75–77). The mechanism whereby this attenuation occurs involves epigenetic programming processes, namely, increased DNA methylation of the gene coding for the glucocorticoid receptor *NR3C1* (78). Epigenetics modifications have also been held responsible for the effects of prenatal maternal stress on gestational length (26); however, whether these pathways include the regulation of glucocorticoid synthesis is unknown.

In the current study, we also report that stress during gestation modestly reduced the litter size in the directly exposed second and third generations, suggesting that fecundity is impacted by maternal stress (79, 80). Yet, all neonates born to stressed dams were viable, suggesting that prenatal maternal stress afflicts reproductive health but does not cause neonatal death. It is worth mentioning that the reduced litter size was not associated with a rise in corticosterone levels. Thus, we suggest that cumulative stress skews the HPA axis in a manner that is not reflected in systemic corticosterone levels. Importantly, we observed that prenatal maternal stress delayed the growth of neonates at week 1. These findings are in line with prior studies showing impaired offspring growth upon maternal stress (26, 81). These data could be explained by the substantial compilation of evidence suggesting that exposure to prenatal stress impacts health and disease susceptibility in the offspring (7–9, 13, 15–17, 82, 83).

Exposure to chronic stress impacts the adaptive immune system (84); thus, we investigated whether prenatal maternal stress alters neonatal adaptive immunity. In this study, we observed a consistent reduction in the pool of naïve and conventional T cells, as well as B cells, in neonates born to F2-SS dams. These findings are in line with previous reports showing that stress induces a decrease in the number of lymphocytes (i.e., lymphopenia) in subsets such as CD4+, CD8+, and B220+ cells (85, 86). A possible mechanism whereby prenatal maternal stress induces neonatal lymphopenia involves the triggering of a fetal systemic inflammatory response that, in turn, may cause involution of the lymphatic organs (87, 88).

Prenatal maternal stress also induced a neonatal immunosuppression-like syndrome characterized by reduced numbers of IL-4- and IL-17-expressing T cells, as well as regulatory CD4+ T cells, in the second generation. All of these T cell subsets have regulatory and anti-inflammatory functions (89–93); therefore, it is likely that prenatal maternal stress alters the T cell repertoire *in utero*. Yet, further research is required to investigate the mechanisms whereby prenatal maternal stress induces immunosuppression in neonates born to mothers and grandmothers who underwent stress during pregnancy.

Notably, we found that the neonatal immunosuppressive-like syndrome induced by prenatal maternal stress was overcome in the third generation. Indeed, neonates born to third generation stressed dams had greater numbers of Th2 cells, IL-4+ CD8+ T cells, and tended to have higher numbers of regulatory CD4+ T cells. To our knowledge, this is the first demonstration to show that chronic exposure to multigenerational stress boosts the neonatal immune system. We ought to propose that this response is observed as a compensatory mechanism against prenatal maternal stress.

Importantly, in the mouse model, the cessation of stress in the second generation restores the timing of delivery. Our results in mice are partially in agreement with those reported in rats; cessation of prenatal maternal stress in rats does not fully restore timing of delivery (26). This discrepancy could be explained by variations in how different species respond to stress (94, 95). Yet, our finding in mice holds tangible and immediate public health relevance given the implication that daughters of stressed mothers may be able to manage the impacts of ancestral stress during their pregnancy. Additional research is warranted to investigate whether the cessation of stress impacts neonatal immunity given that neonatal growth impairment was not restored.

There are some limitations to the current study that we must acknowledge and address. First, using a murine model presents inevitable discrepancies given the fundamental mouse-human differences. However, studying stress in humans is very difficult due to a multitude of variables and uncontrollable co-factors, and can only be done from an epidemiological standpoint. Past studies have demonstrated that efficiently inducing stress in rodents is difficult due to their adaptability (96, 97), but we compensated for this condition by prolonging the period of stress and utilizing an unpredictable, multi-procedural stress schedule. Another limitation of our study is that we utilized a syngenic mating model, which does not allow us to evaluate the contribution of allo-antigenicity; yet, this syngenic model was chosen to maintain prenatal stress as our singular variable. It is worth mentioning that, in this study, we did not include non-stressed controls for every generation; therefore, our experimental design does not allow us to distinguish the transgenerational effects of stress in the second and third generations. An important strength of our study is that we performed two different cohorts of observational studies in which we observed very similar results; both cohorts included animals that delivered preterm. Yet, the execution of this study required a large investment of time and funds as well as the creation of a multidisciplinary team.

## Conclusion

The data presented herein provides a causal link between prenatal maternal stress and preterm birth, as well as neonatal adaptive immunity, across generations. We report that the impact of ancestral prenatal maternal stress results in inheritable consequences, but these alterations can be mitigated by intervention, as well as progressive stress sensitization over time. These findings may hold clinical importance for individuals exposed to direct or ancestral chronic stress and their ability to overcome adverse pregnancy and neonatal outcomes.

## Data Availability Statement

The datasets generated for this study are available on request to the corresponding author.

## Ethics Statement

The animal study was reviewed and approved by the Institutional Animal Care and Use Committee at Wayne State University.

## Author Contributions

VG-F and A-EF performed research and analyzed and interpreted data. RR analyzed and interpreted data and provided guidance in the experimental design. DL, JG, and CZ analyzed and interpreted data. C-DH, SH, DO, and GM provided intellectual input. NG-L designed research, analyzed and interpreted data, and provided supervision throughout the study. All authors participated in the writing of the manuscript.

### Conflict of Interest

The authors declare that the research was conducted in the absence of any commercial or financial relationships that could be construed as a potential conflict of interest.

## Supplementary Material

Supplementary Video 1 Pregnant mice exposed to swimming.

Supplementary Video 2 Pregnant mice exposed to restrain.

Supplementary Video 3 Pregnant mice exposed to shaking.

Supplementary Video 4 Pregnant mice exposed to white noise.

## Abbreviations

ACTH: adrenocorticotropic hormone
BSA: bovine serum albumin
CRH: corticotropic-releasing hormone
dpc: days post coitum
F0-S: pregnant mice stressed during gestation
F1-S: pregnant daughters that were stressed during gestation
F2-SS: pregnant granddaughters that were stressed during gestation
F1-SNS: prenatally stressed pregnant daughters who were not stressed during gestation
HPA: hypothalamic-pituitary-adrenal
IFN: interferon
Ig: immunoglobulin
IL: interleukin
PBS: phosphate-buffered saline.
