# Supplementary material for: Prenatal Maternal Stress Causes Preterm Birth and Affects Neonatal Adaptive Immunity in Mice
Source: Front Immunol. 2020 Feb 26;11:254. doi: 10.3389/fimmu.2020.00254 (PMC7054386; doi:10.3389/fimmu.2020.00254)
Supplement: Supplementary file 5 [file Data_Sheet_1.PDF]

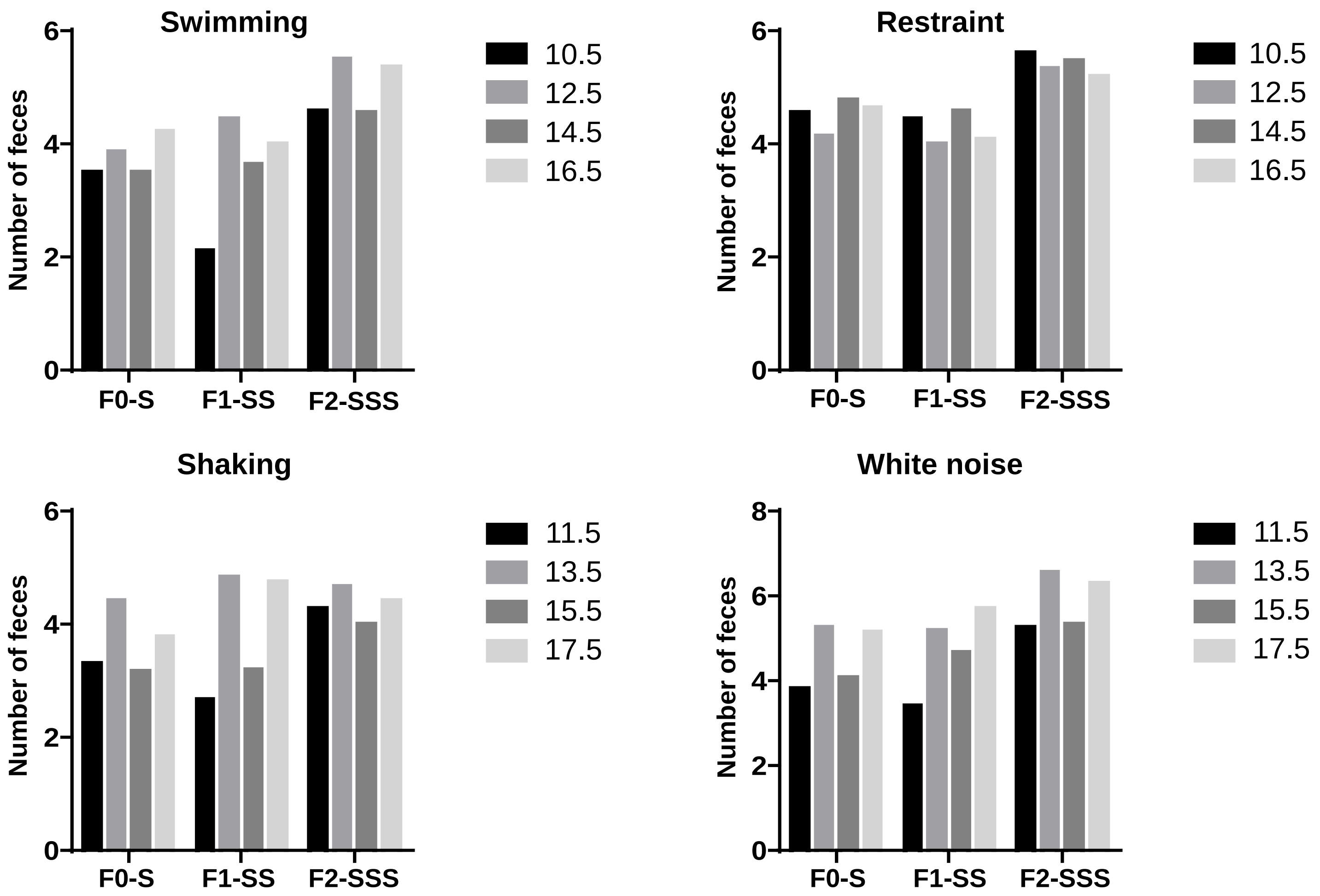

Supplementary figure 1. Quantification of feces during prenatal stress. Feces were counted after the application of prenatal stress induced by swimming, restraint, shaking, or white noise on 11.5, 13.5, 15.5, and 17.5 days *post coitum* (dpc). Feces quantification was performed for each generation of stressed dams. *n* = 8-11 each.
