# Supplementary material for: Prenatal Maternal Stress Causes Preterm Birth and Affects Neonatal Adaptive Immunity in Mice
Source: Front Immunol. 2020 Feb 26;11:254. doi: 10.3389/fimmu.2020.00254 (PMC7054386; doi:10.3389/fimmu.2020.00254)
Supplement: Supplementary file 6 [file Data_Sheet_2.PDF]

Supplementary Table 1. Antibodies used for immunophenotyping.

**Marker**	**Color**	**Clone**	**Catalog #**	**RRID**	**Company**
CD3	APC-Cy7	145-2C11	557596	AB_396759	BD
CD4	PE-Cy5	RPA-T4	555348	AB_395753	BD
CD8	PE-CF594	53-6.7	562283	AB_11152075	BD
CD25	APC	PC61	557192	AB_398623	BD
IL-17A	AF700	TC11-18H10	560820	AB_2034015	BD
IFNɣ	V450	XMG1.2	560661	AB_1727534	BD
IL-4	PE-Cy7	11B11	560699	AB_1727548	BD
FoxP3	AF488	MF23	560403	AB_1645192	BD
CD71	PE	C2	553267	AB_394744	BD
TER 119 (Ly-76)	V450	TER-119	560504	AB_10563222	BD
CD4	APC	RM4-5	553051	AB_398528	BD
CD44	AF700	IM7	560567	AB_1727480	BD
CD62L	PE-Cy7	MEL-14	560516	AB_1645257	BD
CD44 Isotype (Rat IgG2b *κ*)	AF700	A95-1	557964	AB_396964	BD
CD62L Isotype (Rat IgG2a *κ*)	PE-Cy7	R35-95	552784	AB_394465	BD
B220 (CD45R)	APC-Cy7	RA3-6B2	552094	AB_394335	BD
CD5	PE-Cy7	53-7.3	25-0051-81	AB_657755	eBioscience
CD23	PE	B3B4	553139	AB_394654	BD
Fc Block	-	2.4G2	553142	AB_394657	BD
